# Role of the trigeminal mesencephalic nucleus in rat whisker pad proprioception

**DOI:** 10.1186/1744-9081-6-69

**Published:** 2010-11-15

**Authors:** Ombretta Mameli, Stefania Stanzani, Gabriele Mulliri, Rosalia Pellitteri, Marcello A Caria, Antonella Russo, Pierluigi De Riu

**Affiliations:** 1 Department of Neuroscience: Human Physiology Division, University of Sassari, viale San Pietro 43/B, 07100 Sassari, Italy; 2 Department of Physiological Sciences, University of Catania, 95125 Catania, Italy; 3 Institute of Neurological Sciences, National Research Council, Section of Catania, 95125, Catania, Italy

## Abstract

**Background:**

Trigeminal proprioception related to rodent macrovibrissae movements is believed to involve skin receptors on the whisker pad because pad muscles operate without muscle spindles. This study was aimed to investigate in rats whether the trigeminal mesencephalic nucleus (TMnu), which provides proprioceptive feedback for chewing muscles, may be also involved in whisker pad proprioception.

**Methods:**

Two retrograde tracers, Dil and True Blue Chloride, were injected into the mystacial pad and the masseter muscle on the same side of deeply anesthetized rats to label the respective projecting sensory neurons. This double-labeling technique was used to assess the co-innervation of both structures by the trigeminal mesencephalic nucleus (TMnu).

In a separate group of anesthetized animals, the spontaneous electrical activities of TMnu neurons were analyzed by extracellular recordings during spontaneous movements of the macrovibrissae. Mesencephalic neurons (TMne) were previously identified by their responses to masseter muscle stretching. Changes in TMne spontaneous electrical activities, analyzed under baseline conditions and during whisking movements, were statistically evaluated using Student's *t*-test for paired observations.

**Results:**

Neuroanatomical experiments revealed different subpopulations of trigeminal mesencephalic neurons: i) those innervating the neuromuscular spindles of the masseter muscle, ii) those innervating the mystacial pad, and iii) those innervating both structures. Extracellular recordings made during spontaneous movements of the macrovibrisae showed that whisking neurons similar to those observed in the trigeminal ganglion were located in the TMnu. These neurons had different patterns of activation, which were dependent on the type of spontaneous macrovibrissae movement. In particular, their spiking activity tonically increased during fan-like movements of the vibrissae and showed phasic bursting during rhythmic whisking. Furthermore, the same neurons may also respond to masseter muscle stretch.

**Conclusions:**

results strongly support the hypothesis that the TMnu also contains first-order neurons specialized for relaying spatial information related to whisker movement and location to trigeminal-cortical pathways. In fact, the TMnu projects to second-order trigeminal neurons, thus allowing the rat brain to deduce higher-order information regarding executed movements of the vibrissae by combining touch information carried by trigeminal ganglion neurons with proprioceptive information carried by mesencephalic neurons.

## Background

Previous studies have shown that extratrigeminal fibers in the rat, originating in the hypoglossal nucleus and traveling along the infraorbital division of the trigeminal nerve, join the ipsilateral muzzle. These hypoglossal terminals, which target the extrinsic muscles surrounding the mystacial follicles of the macrovibrissae [1,2], significantly modulate the electromyographic activity of the whisker pad motor units [2]. Furthermore, it has been demonstrated that hypoglossal innervation also extends to the masticatory masseter muscle. In particular, the hypoglossal terminals target the polar regions of the intrafusal fibers of a number of the masseter neuromuscular spindles [3]. Taken together, these findings demonstrate that motor control of the whisker pad structures performed by known facial and trigeminal motor neurons also involves the hypoglossal nucleus, which appears to incorporate them into the same functional unit. On the other hand, extensive projections from sensory trigeminal nuclei enclosed in the mesencephalic trigeminal nucleus to the hypoglossus have been largely described [see [4] for a review], although their functional significance warrants further inquiry.

The common motor innervation of the muzzle structures by the hypoglossal nucleus [5] suggests the possibility of a similar functional arrangement with respect to their sensory innervation. In the rat, sensory signals from whisker-pad structures are conducted by trigeminal ganglion neurons to trigeminal sensitive nuclei [[6-8]; see [4], [9-11] for a review] and from the masticatory muscles also by trigeminal mesencephalic neurons (TMne). The latter possess neuromuscular spindles, which are absent from whisker pads [12], to provide sensory feedback for masticatory muscle proprioception [13,5]. The present study was aimed at investigating the hypothesis that the mesencephalic trigeminal nucleus (TMnu) may provide proprioceptive feedback for the whisker-pad structures as well.

## Materials and methods

The experiments were performed on Wistar rats weighing 250-300 g (Morini, San Polo D'Enza, Italy). This species was chosen based on previous studies by this and other groups [14,15,1-3].

In accordance with current institutional guidelines for the care and use of experimental animals, laboratory chow and water were available *ad libitum*, and animals were housed under controlled conditions (room temperature: 23 ± 1°C; lights on: 07.00 to 19.00). All experimental procedures were approved by the Italian Health Ministry and the local Veterinary Public Health Service.

### Neuroanatomical procedures

For tract-tracing studies, animals (*n *= 8) were first deeply anesthetized (sodium pentobarbital, 75 mg/kg^-1^, intraperitoneal [i.p.]). Under aseptic conditions, the tracer Dil (1,1'-dioctadecyl 3,3,3',3'-tetramethyl-indocarbocyanine perchlorate, Molecular Probes, Inc.) was unilaterally injected into the mystacial pad by four injections (0.05 μL each) able to mark all the whisker muscles and to label the sensory neurons projecting to the pad in a retrograde manner. Tracer injections were always performed near the same whiskers to label the same structures in each rat.

True Blue Chloride was also injected into the ipsilateral masseter muscle by six injections (0.05 μL each) into different sites of the same animals to mark all muscles and label sensory-projection neurons. Each tracer was injected by positive pressure at 50 nL/min using a Hamilton micro-syringe (1 μL). The double labeling technique was used to assess a possible co-innervation of both structures by the TMnu, which are known to provide sensory innervation to the masseter muscle spindles [13,5].

Seven days following tracer injections, the animals were again deeply anaesthetized (sodium pentobarbital, 75 mg/kg, intraperitoneal [i.p.]) and transcardially perfused through the ascending aorta with 200-250 mL of saline, followed by 200-250 mL of ice-cold 4% paraformaldehyde in 0.1 M phosphate-buffered saline (PBS, pH 7.4). Their brains were rapidly removed, post-fixed for 3-4 h, and cryoprotected overnight in phosphate-buffered 20% sucrose solution. The brainstem was sectioned in the transverse plane using a freezing microtome (40-μm thickness). Serial sections were collected in phosphate buffer, mounted on slides, and observed under a Nikon Eclipse 80i fluorescence microscope. Selective filters for True Blue (360-nm wavelength) and Dil (560-nm wavelength) were used, and the images were recorded with a digital camera (Nikon).

### Electrophysiological procedures

A separate group of animals (*n *= 10) was used to analyze the electrical activities of the trigeminal mesencephalic neurons (TMne) during spontaneous movements of their macrovibrissae. The mystacial pad macrovibrissae, easily distinguishable from the more anterior microvibrissae [14], are long tactile hairs in the animal's muzzle that are laterally oriented, aligned in regular rows, and arranged in a precise dorsoventral pattern [16,17].

In these experiments, animals were anesthetized by i.p. injection of diazepam (30 mg/kg) and ketamine hydrochloride (45 mg/kg) and placed in a stereotaxic frame. The ECG was continuously monitored throughout the experiment to assess the depth of anesthesia and animal discomfort. Following craniotomy, the cerebellum and obex were exposed at the level of the parietal and occipital bones. A laminectomy at C_1_-C_2 _levels completed the surgical procedures. All exposed surfaces were then protected with warm mineral oil and paraffin (37°C) while the pressure points and the surgical area were injected with xylocaine (0.3%) every 40 min.

When surgical manipulations were terminated, macrovibrissae movement was carefully monitored. In anesthetized and non-paralyzed animals, the macrovibrissae are usually motionless; however, spontaneous movement of single or multiple macrovibrissae can occur infrequently and can continue for several minutes.

Spontaneous whisker movements show certain characteristic patterns. In particular, we noted and analyzed two conditions: i) rhythmic whisking, characterized by sweeping forward and backward movements of the whiskers, and ii) a slow fan-like opening of the whisker bundle to disseminate the tips of the vibrissae to explore the space surrounding the sides of the animal muzzle. In every case, these movements were always followed by a slow return of the vibrissae to the resting position.

Although the whisker movements did not provide precise information because exact recordings were not conducted, they seemed to cover approximately 80-100°.

The spontaneous electrical activity of trigeminal mesencephalic neurons (TMne), previously identified by their responses to masseter muscle stretching, was extracellularly recorded using tungsten-in-glass microelectrodes (impedance = 700-1,200 KΩ) slowly advanced into the TMnu using an electronic microdrive (David Kopf).

The electrical signals were relayed to conventional preamplifiers and then passed to a computer and analyzed with specific software (Tecfen computerscope analysis ISC-16 software, and PowerLab 4/30 Chart 5, V 5.4.2 software). The spontaneous electrical activity of single TMne and their responses to stretching of the masseter muscle were analyzed. Recordings were carried out under baseline conditions (i.e., motionless) and repeated during the whisker movements described above.

At the conclusion of the experiment, the recording site was marked by an electrolytic lesion (20 μA, 20 s, cathodal current), and the animal was sacrificed by barbiturate overdose. The brain was removed, fixed in Carnoy's solution, and embedded in paraffin for subsequent histological procedures. Serial sections (40-μm thick) of the brainstem were cut and stained with cresyl violet to localize the recording sites.

### Statistical analysis procedures

Changes in TMne spontaneous electrical activity, analyzed under baseline conditions and during both types of whisking movement, were statistically evaluated using Student's *t*-test for paired observations.

## Results

### Neuroanatomical findings

Following tracer injections into the mystacial pad and the ipsilateral masseter muscle, neurons labeled with both tracers were detected in the trigeminal mesencephalic nucleus (TMnu). One hundred neurons were studied in each animal.

**Figure 1:**
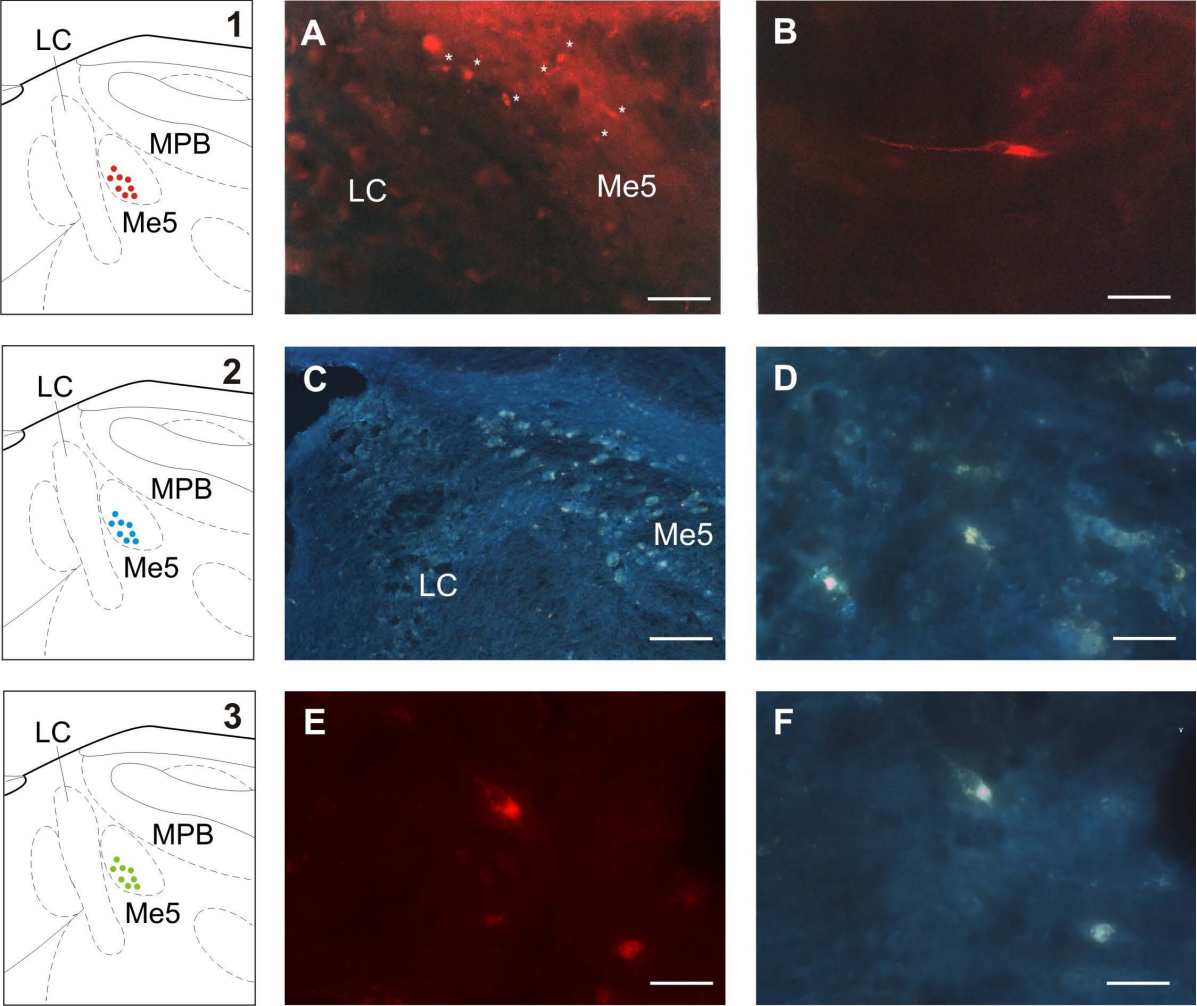
**Labeled trigeminal mesencephalic neurons following tracer injections into the vibrissal pad (Dil) and masseter muscle (True Blue)**. **1-3: **Schematic drawings of brainstem sections at the caudal level of the mesencephalic trigeminal nucleus (Me5) showing the locations on the cell bodies of axons joining the whisker pad (1: red dots), the masseter muscle spindles (2: blue dots), and both structures (3: green dots). LC: locus ceruleus; MPB: medial parabrachial nucleus. **A: **Labeled neurons (asterisks) within the Me5 following Dil injection into the ipsilateral mystacial pad. Scale bar: 50 μm. **B: **Greater magnification of a trigeminal mesencephalic neuron (TMne) labeled by Dil. Scale bar: 10 μm. **C: **Labeled neurons in the Me5 following True Blue injection into the ipsilateral masseter muscle. Scale bar: 100 μm. **D: **Greater magnification of two TMne labeled by True Blue. Scale bar: 20 μm. **E-F: **Greater magnification of a TMne double labeled by Dil (E) and True Blue (F). Scale bar: 20 μm.

Histological analysis showed that mesencephalic neurons labeled by Dil had a fusiform shape, with a mean longitudinal diameter of 10.20 μm (Figure 1A, B). Mesencephalic neurons labeled by True Blue were instead more oval in shape and larger in size compared to those labeled by Dil, and they had a mean longitudinal diameter of 20.4 μm (Figure 1C, D). Finally, some TMne, similar in shape to the others but with a mean longitudinal diameter of 22.4 μm, (Figure 1E-F) showed double labeling for both tracers. More specifically, an average of 30% of the analyzed neurons labeled by Dil projected to the mystacial pad, 60% labeled by True Blue projected to the masseter muscle, and the remaining 10% that were double labeled projected to both structures.

All neurons were localized in the medio-caudal part of the mesencephalic nucleus (from -9.68 to -10.04 mm posterior to bregma [18]) without any specific segregation by tracer label (Figure 1 drawings 1-3).

### Electrophysiological findings

The spontaneous electrical activities of 65 TMnu neurons were studied and recorded during spontaneous movements of the macrovibrissae and under resting conditions.

As previously described in the methods section, we examined two patterns of vibrissae movement: rhythmic whisking and fan-like divarication. The trigeminal mesencephalic neurons located in the medio-caudal part of the nucleus (from -9.68 to -10.04 mm posterior to bregma; [18]) responded to the stretching of the masseter muscle as well as to the spontaneous movement of the macrovibrissae. Thirty-nine neurons showed this pattern of response.

**Figure 2:**
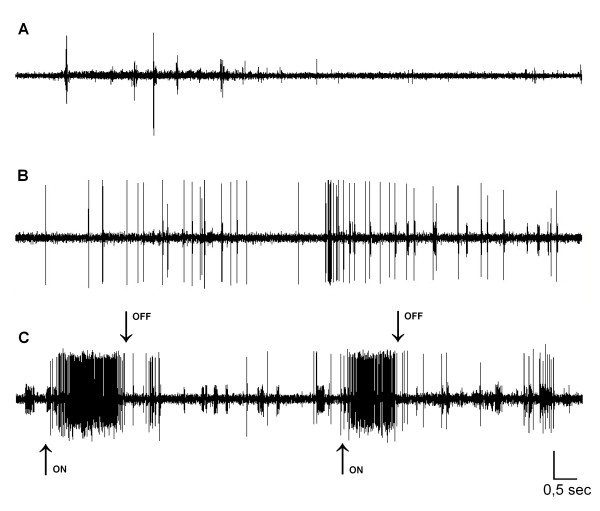
**Spontaneous electrical activity of a trigeminal mesencephalic neuron responsive to fan-like movement of the macrovibrissae bundle and to masseter muscle stretching**. **(A**) Spontaneous electrical activity of a TMne under baseline conditions (i.e., with the vibrissae in a resting position). **(B) **The same activity recorded during fan-like movement of the macrovibrissae bundle and **(C) **during both fan-like whisker movements and masseter muscle stretching. "On" and "off" arrows show the start and the stop of the masseter stretch, respectively. Calibrations: horizontal 0.5 s; vertical 250 μV.

During fan-like divarication of the macrovibrissae, the TMne showed a significant increase in spontaneous firing rates compared to those under baseline conditions (*P *< 0.001). These fan-like movements were often repeated several times, with each cycle lasting several seconds. When movement stopped, TMne firing returned to baseline values. Figure 2 shows a representative trigeminal mesencephalic neuron that responded to this type of macrovibrissae movement as well as to stretching of the masseter muscle.

**Figure 3:**
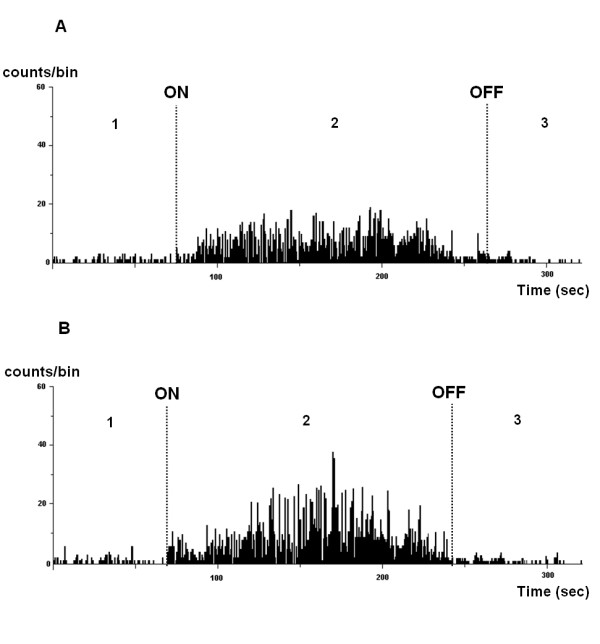
**Spontaneous electrical activity of a trigeminal mesencephalic neuron recorded under the resting condition of the vibrissae and during their spontaneous movement**. **(A) **Frequency distribution histogram showing the spontaneous electrical activity of a trigeminal mesencephalic neuron (TMne) recorded while the macrovibrissae were under the resting condition (1); during their spontaneous movements, which lasted 3.83 min in this instance (2); and at the end of the movement, when the vibrissae returned to the resting condition (3). **(B) **The histogram shows the behavior of the same TMne recorded before (1), during (2), and after (3) vibrissae displacements during another episode of vibrissae whisking, which lasted 3.25 min in this case. In both histograms, "ON" and "OFF" indicate the start and the end, respectively, of the vibrissae displacement. The discharge frequency of the neuron (counts/bin) followed the increase or the decrease of the vibrissae displacement. Horizontal calibration: 50 sec, vertical calibrations: 10 counts/bin.

**Figure 4:**
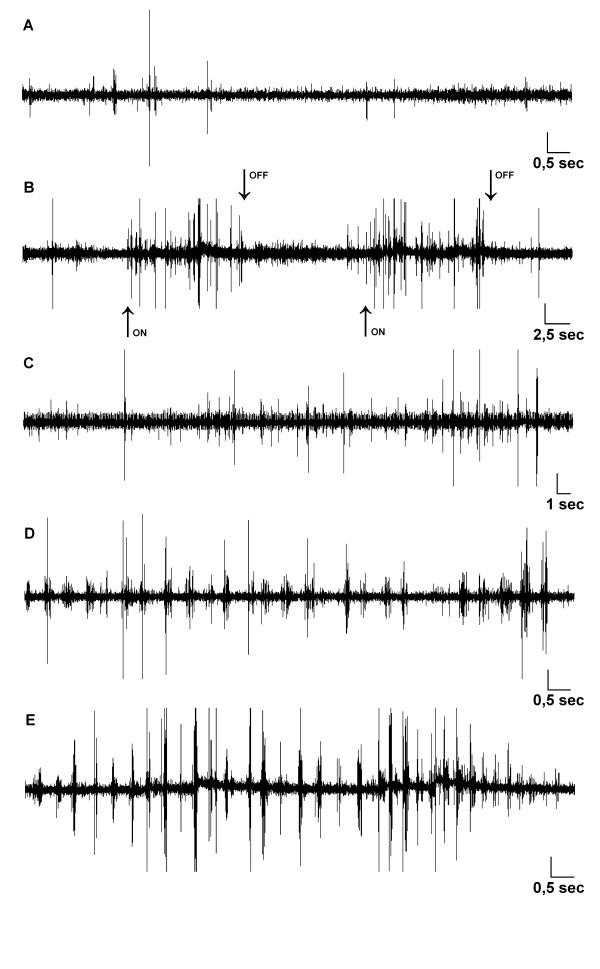
**Spontaneous electrical activity of a trigeminal mesencephalic neuron responsive to spontaneous macrovibrissae movement and masseter stretching**. **(A) **Spontaneous electrical activity of a trigeminal mesencephalic neuron under basal conditions (i.e., with vibrissae in resting position). (**B) **The same activity during the masseter stretch. Arrows indicate the beginning ("on") and the end ("off") of muscle stretching. **(C) **The same activity during fan-like movement of the macrovibrissae bundle and (**D, E) **during rhythmic whisking movements at different frequencies. Calibrations: horizontal, indicated in picture; vertical 250 μV for all recordings.

During rhythmic whisking, the TMne discharge pattern significantly changed, becoming less regular, with phasic bursts that corresponded to whisker motion. The burst frequency significantly increased or decreased in direct relation to the increase or decrease, respectively, of vibrissae displacement. Rhythmic whisking lasted up to several seconds (3-5 sec), and when vibrissae movement ceased, burst firing also stopped, and TMne activity reverted to a tonic firing pattern, slowly returning to the baseline firing rate (Figure 3). Figure 4 shows a representative TMne that responded to the masseter muscle stretch (Figure 4B) and to both types of macrovibrissae movements (Figure 4C-E).

**Figure 5:**
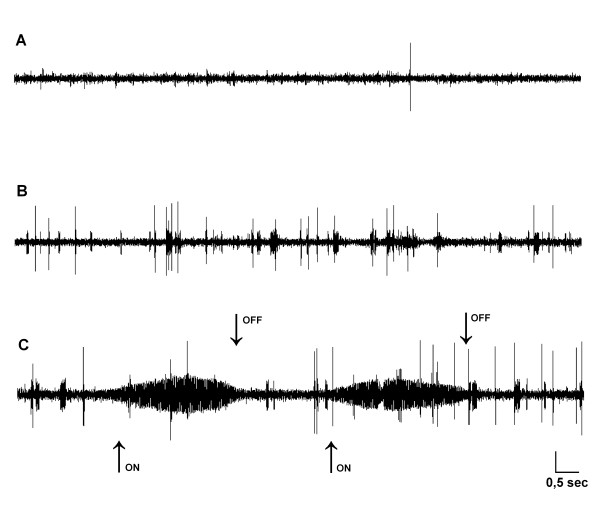
**Spontaneous electrical activity of a trigeminal mesencephalic neuron responsive only to spontaneous movement of the macrovibrissae bundle**. **(A) **Spontaneous electrical activity of a trigeminal mesencephalic neuron under baseline conditions (i.e., with the vibrissae in resting position). **(B) **The same activity during fan-like movement of the macrovibrissae bundle and **(C) **during simultaneous stretching of the masseter muscle. The neuron did not respond to the masseter stretch, which only induced evoked field potentials in the nucleus. Arrows indicate the beginning ("on") and the end ("off") of the muscle stretching. Calibrations: horizontal 0.5 s; vertical 250 μV.

Finally, some mesencephalic neurons responded only to the masseter stretch whereas others responded only to the spontaneous macrovibrissae movement. In particular, we analyzed 14 neurons that responded only to the masseter stretch and 12 that responded only to the spontaneous movement of the macrovibrissae. Figure 5 shows the spontaneous electrical activity of a representative TMne that responded only to macrovibrissae movement (Figure 5B, C). The masseter muscle stretch, applied during fan-like movement of the macrovibrissae, induced only an evoked potential in the nucleus without changing the neuronal spiking activity (Figure 5C).

## Discussion

A number of behavioral studies have shown that rats can use their macrovibrissae to locate an object in the environment and detect its position in relation to the position of its own head [19]. Signals from whiskers contacting an object can be integrated to calculate the object's radial distance [20,21] and the object features in the vertical plane through trigeminal ganglion neurons that provide vertical coordinates of the object [19]. The radial location of an object is thought to be encoded by touch cells and whisking/touch cells of the trigeminal ganglion via changes in firing rates and/or spike counts [10,11,22], whereas it has been proposed that horizontal location is encoded using the same sensory signal but relying instead on spike timing rather than on spike counts. Alternatively, the spiking activity of whisking cells in the trigeminal ganglion may provide a reference signal [11] that encodes the spatial location of the whisker over time [23].

It has been proposed that rats may locate an object and define its coordinates by combining contact-evoked information from peripheral receptors with internally generated motor signals. Thus, to learn about an executed movement, sensory pathways may receive copies of the motor signal from the brainstem, zona incerta, cerebellum, and motor cortices [24-28]. Alternatively, the sensory pathway itself may carry afferent signals, through the whisking cells observed in the trigeminal ganglion and thalamic nuclei, regarding the whisker motion resulting from contacting an object [10,11,29] horizontal position may be encoded by neuronal spike counts [30-32]. Recently, Knutsen and Ahissar [33] reviewed evidence that the rodent vibrissal system encodes the three-dimensional location of objects by means of an orthogonal triple-code scheme. Neural recordings from primary sensory afferents, along with behavioral observations, demonstrate that the vertical coordinates of contacted objects are encoded by the identity of activated afferents, horizontal coordinates by the timing of activation, and radial coordinates by the intensity of activation.

Thus, it is clear that encoding of whisker motion relies on a sophisticated mechanism that is essential for rat spatial orientation and perception. However, vibrissae motion apparently lacks a peripheral control mechanism, as it has been reported that pad muscles appear to operate without feedback control from muscle spindles [12]. It has been suggested that skin receptors may be involved in proprioception [34,35], as during vibrissae whisking, and coherent oscillatory spike activity has been observed in the principal and spinal trigeminal nuclei [36,37,10,11]. A significant increase in 2-deoxyglucose uptake during vibrissae movements evoked by stimulation of the rat motor cortex has been described in discrete subcortical regions, including the trigeminal spinal nuclei, which have been suggested as providing proprioceptive input to the sensorimotor cortex as a result of vibrissae movement [38]. Rats may therefore infer the position of their vibrissae through sensory afferents projecting to the trigeminal sensitive nuclei.

We believe that the present findings provide novel insights regarding sensory control of the mystacial pad. Our results demonstrated that whisking neurons similar to those described in the trigeminal ganglion are located in the TMnu [39,10,11,42] and that spontaneous macrovibrissae movement produced significant changes in the spontaneous electrical activity of TMne. In particular, spiking activity tonically increased during fan-like movements of the vibrissae and showed phasic bursting during rhythmic whisking. Furthermore, the same neurons also responded to masseter muscle stretching, which is consistent with our neuroanatomical results showing the existence of double-labeled TMne simultaneously projecting to the mystacial pad and to the masseter muscle.

It seems appropriate to assign a proprioceptive function to the TMne terminals located in the mystacial pad in view of the following observations. During neuromuscular spindle activation, the TMne encodes spatial information related to mechanical distortion of masseter muscle cells [43,13,5]. Therefore, it is plausible that the TMnu neurons simultaneously innervating both the masseter neuromuscular spindles and the mystacial pad may serve the same proprioceptive function with respect to the mystacial pad despite the absence of spindles in the latter. A similar double representation of primary sensory neurons in the trigeminal ganglion and the TMnu has already been described with regard to the periodontal ligament [[44,45]; see [46] for a review], although it has been believed for many years that both were mechanoreceptors. More recent studies have raised some doubts about this interpretation and concluded that periodontal ligament receptors with cell bodies in the TMnu may play a different functional role from at played by those with cell bodies in the TMnu [47]. In our opinion, these results on the sensory system of the macrovibrissae suggest that this functional hypothesis should be investigated.

Our findings also show that neurons in the TMnu responded only to spontaneous movement of the vibrissae in that mandibular stretching induced only remote potentials in the nucleus, demonstrating that TMne responses were not necessarily related to parallel jaw movements. Thus, vibrissae movement may induce mechanical distortion of TMne peripheral terminals with consequent stimulation. Experiments in progress are aimed at identifying the TMne peripheral terminals within the mystacial pad to verify whether they target its extrinsic and/or intrinsic muscles or the follicle-sinus complex. In any case, it appears that the TMnu is involved in encoding spatial information related to vibrissae movement. This hypothesis is further supported by the presence of labeled TMne innervating the mystacial pad as well as by our electrophysiological data, which characterize neurons that specifically encode vibrissal spatial information.

### Limitations

It is known that, lacking in neuromuscular spindles, the whisker pad muscles appear to operate without their feedback control. The present results suggested that skin receptors, represented by peripheral axon terminals of the trigeminal mesencephalic nucleus, may be involved in sensory proprioception, allowing spatial coding regarding the executed movements of the vibrissae. With concern the sensorimotor loop, it has been proposed that hypoglossal nucleus may be involved in the motor control of the mystacial pad-masseter muscle complex in response to the sensory TMnu afferents from these same structures. However, this hypothesis must be evaluated to further clarify the complex sensory motor control of the oro-facial system.

## Conclusions

Neuroanatomical findings showed that neurons labeled with both tracers were detected in the trigeminal mesencephalic nucleus (TMnu) following tracer injection into the mystacial pad and the ipsilateral masseter muscle. These observations are consistent with electrophysiological results showing that TMNe exhibited significant changes in firing rates during spontaneous movements of the macrovibrissae, which were characterized by fan-like divarication as well as by rhythmic whisking. On the other hand, when movement stopped, TMne firing returned to baseline values.

Taken together, these findings strongly support the idea that the TMnu includes first-order neurons specialized for conveying spatial information related to whisker location to trigeminal-cortical pathways.

In particular, results suggest that the rat brain may deduce higher-order information regarding executed movements of the vibrissae by combining touch information, carried by trigeminal ganglion neurons, with proprioceptive information, carried by mesencephalic whisking neurons. The central projection of the TMne primarily subserves masticatory reflexes, but projections to the other trigeminal nuclei, which may have a sensory function, may also exist [see [48] for a review]. The TMnu projects to the principal trigeminal nucleus [49-51] and therefore, its *barrelettes*, which receive touch information from the trigeminal ganglion neurons, may integrate this information with proprioceptive feedback received from TMne. Convergence of these sensory two modalities could provide a more detailed definition when reconstructing the surrounding environment. Furthermore, the important role of the cerebellum must also be considered with respect to the fine control of vibrissae movements and the detection of their spatial locations, which, in our opinion, cannot be restricted to merely transferring copies of motor signals to sensory pathways, as proposed by some authors [24-28]. Aside from the afferents from all trigeminal sensory nuclei [52], the cerebellar cortex receives significant afferents from the caudal part of the TMnu [52,53], the same TMnu portion that, as shown by the present results, also includes the cell bodies of whisker receptors. In this context, the TMnu may represent the crucial relay for the cerebellar proprioceptive perception of the vibrissae system.

An intriguing question raised by these findings relates to the motor circuitry by which the TMnu may induce reflex responses in the whisking-mastication muscle complex. To date, it has been thought that masticatory muscles possess only a proprioceptive reflex that is mediated by trigeminal motor neurons. However, it has been shown that axons of the TMne also target the ipsilateral hypoglossal nucleus, and proprioceptive afferents from the masseter muscle directly project to the hypoglossal nucleus [54-57]. Hypoglossal efferences have been also demonstrated in the extrinsic muscles of the mystacial pad and in masseter neuromuscular spindles [2,3]. Therefore, the hypoglossal nucleus may be involved in the motor control of the mystacial pad-masseter muscle complex in response to the sensory TMnu afferents from these same structures. This hypothesis is currently being evaluated to further clarify the complex sensorimotor control of the oro-facial system.

## List of abbreviations

**TMnu: trigeminal mesencephalic nucleus; TMne: trigeminal mesencephalic neurons**.

## Competing interests

The Authors declare there is not any financial or non-financial competing interest that might influence the results or interpretation of the enclosed paper.

## Authors' contributions

Conception, design, analysis and interpretation data were performed by OM and SS, and acquisition data was carried out also by GM, RP and AR. The revising of the manuscript for important intellectual content was performed by MAC and PLD. All authors read and approved the final manuscript, which was prepared by OM.

## References

[B1] Mameli O Pellitteri R Russo A Stanzani S Caria MA De Riu PL Role of trigeminal nerve in regrowth of hypoglossal motoneurons after hypoglossal-facial anastomosis Acta Oto-Laryngol 2006 126 1334 1338 10.1080/00016480600801332 17101597

[B2] Mameli O Stanzani S Russo A Romeo R Pellitteri R Spatuzza M Caria MA De Riu PL Hypoglossal nuclei participation in rat mystacial pad control Pflügers Arch-Eur J Physiol 2008 456 1189 1198 10.1007/s00424-008-0472-y 18301914

[B3] Mameli O Stanzani S Russo A Pellitteri R Spatuzza M Caria MA Mulliri G De Riu PL Hypoglossal nucleus projections to the rat masseter muscle Brain Res 2009 1283 34 40 10.1016/j.brainres.2009.06.004 19523459

[B4] Waite PME Tracey DJ Paxinos G Trigeminal sensory system The rat nervous system 1995 2 San Diego: Academic Press 705 724

[B5] Lingenhöhl K Friauf E Sensory neurons and motoneurons of the jaw-closing reflex pathway in rats: a combined morphological and physiological study using the intracellular horseradish peroxidase technique Exp Brain Res 1991 83 385 396 10.1007/BF00231163 1708725

[B6] Clarke WB Bowsher D Terminal distribution of primary afferent trigeminal fibers in the rat Exp Neurol 1962 6 372 383 10.1016/0014-4886(62)90019-5 14021584

[B7] Torvik A Afferent connections to the sensory trigeminal nuclei, the nucleus of the solitary tract and adjacent structures: an experimental study in the rat J Comp Neurol 1956 106 51 141 10.1002/cne.901060104 13398491

[B8] Travers JB Norgren R Afferent projections to the oral motor nuclei in the rat J Comp Neurol 1983 220 280 298 10.1002/cne.902200303 6315785

[B9] Travers JB Paxinos G Oromotor nuclei The rat nervous system 1995 2 San Diego: Academic Press 239 255

[B10] Szwed M Bagdasarian K Ahissar E Encoding of vibrissal active touch Neuron 2003 40 621 630 10.1016/S0896-6273(03)00671-8 14642284

[B11] Szwed M Bagdasarian K Blumenfeld B Barak O Derdikman D Ahissar E Responses of trigeminal ganglion neurons to the radial distance of contact during active vibrissal touch J Neurophysiol 2006 95 791 802 10.1152/jn.00571.2005 16207785

[B12] Rice FL Fundin BT Pfaller K Arvidsson J The innervation of the mystacial pad in the adult rat studied by anterograde transport of HRP conjugates Exp Brain Res 1994 99 233 246 7523174 10.1007/BF00239590

[B13] Lund JP Olsson KA The importance of reflexes and their control during jaw movement Trends in Neurosci 1983 6 458 463 10.1016/0166-2236(83)90219-9

[B14] Brecht M Preilowski B Merzenich MM Functional architecture of the mystacial vibrissae Behav Brain Res 1997 84 81 97 10.1016/S0166-4328(97)83328-1 9079775

[B15] Kleinfeld D Berg RW O'Connor SM Anatomical loops and their electrical dynamics in relation to whisking by rat Somatosens & Motor Res 1999 16 69 88 10.1080/08990229970528 10449057

[B16] Dörfl J The innervation of the mystacial region of the white mouse. A topographical study J Anat 1985 142 173 184 17103584 PMC1166371

[B17] Erzurumlu RS Killackey HP Efferent connections of the brainstem trigeminal complex with the facial nucleus of the rat J Comp Neurol 1979 188 75 86 10.1002/cne.901880107 500855

[B18] Paxinos G Watson C The rat brain in stereotaxic coordinates 1997 New York, London, Sydney, Tokyo, Toronto, San Diego: Academic Press, Inc

[B19] Diamond ME von Heimendahl M Knutsen PM Kleinfeld D Ahissar E "Where" and "what" in the whisker sensorimotor system Nature Review Neurosci 2008 9 601 612 10.1038/nrn2411 18641667

[B20] Krupa DJ Matell MS Brisben AJ Oliveira LM Nicolelis MA Behavioral properties of the trigeminal somatosensory system in rats performing whisker-dependent tactile discriminations J Neurosci 2001 21 5752 5763 11466447 10.1523/JNEUROSCI.21-15-05752.2001 PMC6762640

[B21] Schuler MG Krupa DJ Nicolelis MAL Integration of bilateral whisker stimuli in rats: role of the whisker barrel cortices Cereb Cortex 2002 12 86 97 10.1093/cercor/12.1.86 11734535

[B22] Solomon JH Hartmann MJ Biomechanics: Robotic whiskers used to sense features Nature 2006 443 525 525 10.1038/443525a 17024083

[B23] Metha SB Kleinfeld D Frisking the whiskers: patterned sensory input in the rat vibrissa system Neuron 2004 41 181 184 10.1016/S0896-6273(04)00002-9 14741099

[B24] Berg RW Kleinfeld D Rhythmic whisking by rat: retraction as well as protraction of the vibrissae is under active muscular control J Neurophysiol 2003 89 104 117 10.1152/jn.00600.2002 12522163

[B25] Kleinfeld D Ahissar E Diamond ME Active sensation: insights from the rodent vibrissa sensorimotor system Curr Opin Neurobiol 2006 16 435 444 10.1016/j.conb.2006.06.009 16837190

[B26] Hentschke H Haiss F Schwarz C Central signals rapidly switch tactile processing in rat barrel cortex during whisker movements Cereb Cortex 2006 16 1142 1156 10.1093/cercor/bhj056 16221924

[B27] Ahrens KF Kleinfeld D Current flow in vibrissa motor cortex can phase-lock with exploratory rhythmic whisking in rat J Neurophysiol 2004 92 1700 1707 10.1152/jn.00020.2004 15331651

[B28] Veinante P Deschènes M Single-cell study of motor cortex projections to the barrel field in rats J Comp Neurol 2003 464 98 103 10.1002/cne.10769 12866130

[B29] Yu C Derdikman D Haidarliu S Ahissar E Parallel thalamic pathways for whisking and touch signals in the rat PLoS Biol 2006 4 e124 10.1371/journal.pbio.0040124 16605304 PMC1436027

[B30] Ahissar E Arieli A Figuring space by time Neuron 2001 32 185 201 10.1016/S0896-6273(01)00466-4 11683990

[B31] Ahissar E Sosnik R Haidarliu S Transformation from temporal to rate coding in the somatosensory thalamocortical pathway Nature 2000 406 302 306 10.1038/35018568 10917531

[B32] Ahissar E Zacksenhouse M Temporal and spatial coding in the rat vibrissal system Prog Brain Res 2001 130 75 88 full_text 11480290 10.1016/s0079-6123(01)30007-9

[B33] Knutsen PM Ahissar E Orthogonal coding of object location Trends in Neurosci 2008 32 101 109 10.1016/j.tins.2008.10.002 19070909

[B34] Fee MS Mitra PP Kleinfeld D Central versus peripheral determinates of patterned spike activity in rat vibrissa cortex during whisking J Neurophysiol 1997 78 1144 1149 9307141 10.1152/jn.1997.78.2.1144

[B35] Gandieva SC Burke D Cordo P, Harnard S Does the nervous system depend on kinaesthetic information to control natural limb movements Motor contro 1994 New York, Cambridge: University Press 12 30

[B36] Nicolelis MA Ghazanfar AA Faggin BM Votaw S Oliveira LM Reconstructing the engram: simultaneous, multisite, many single neuron recordings Neuron 1977 18 529 537 10.1016/S0896-6273(00)80295-0 9136763

[B37] Nicolelis MAL Baccala LA Lin RCS Chapin JK Sensorimotor encoding by synchronous neural ensemble activity at multiple levels of the sensorymotor system Science 1995 268 1353 1358 10.1126/science.7761855 7761855

[B38] Sharp FR Evans K Regional (^14^C) 2-deoxyglucose uptake during vibrissae movement evoked by rat motor cortex stimulation J Comp Neurol 1982 208 255 287 10.1002/cne.902080305 7119161

[B39] Lichtenstein SH Carvell GE Simons DJ Responses of rat trigeminal ganglion neurons to movements of vibrissae in different directions Somatosens & Motor Res 1990 7 47 65 10.3109/08990229009144697 2330787

[B40] Pali J Rencz B Hamori J Innervation of a single vibrissa in the whisker-pad of rat Neuroreport 2000 11 849 851 10.1097/00001756-200003200-00038 10757532

[B41] Leiser S Moxon KA Response properties of rat trigeminal ganglion neurons during natural whisking behaviors Neuron 2007 53 117 133 10.1016/j.neuron.2006.10.036 17196535

[B42] Khatri V Bermejo R Brumberg JC Keller A Zeigler HP Whisking in air: encoding of kinematics by trigeminal ganglion neurons in awake rats J Neurophysiol 2009 101 1836 1846 10.1152/jn.90655.2008 19109457 PMC2695634

[B43] Brodal A Neurological Anatomy in Relation to Clinical Medicine 1981 New York: Oxford University Press

[B44] Byers MR O'Connor TA Martin RF Dong WK Mesencephalic trigeminal sensory neurons of cat: axon pathways and structure of mechanoreceptive endings in periodontal ligament J Comp Neurol 1986 250 181 191 10.1002/cne.902500205 3745510

[B45] Shigenaga Y Doe K Suemune S Mitsuhiro Y Tsuru K Otani K Shirana Y Hosoi M Yoshida A Kagawa K Physiological and morphological characteristics of periodontal mesencephalic trigeminal neurons in the cat--intra-axonal staining with HRP Brain Res 1989 505 91 110 10.1016/0006-8993(89)90119-4 2611682

[B46] Linden RWA Scott BJJ Distribution of mesencephalic nucleus and trigeminal ganglion mechanoreceptors in the periodontal ligament of the cat J Physiol 1989 410 35 44 2795482 10.1113/jphysiol.1989.sp017519 PMC1190465

[B47] Linden RWA Millar BJ Halata Z A comparative physiological and morphological study of periodontal ligament mechanoreceptors represented in the trigeminal ganglion and the mesencephalic nucleus of the cat Anat Embryol 1994 190 127 135 10.1007/BF00193410 7818086

[B48] Darian-Smith I Iggo A The trigeminal system Handbook of Sensory Physiology (vol. II). Somatosensory System 1973 Berlin, Heidelberg, New York: Springer-Verlag 271 314

[B49] Luo P Wong R Dessem D Projection of jaw-muscle spindle afferents to the caudal brainstem in rats demonstrated using intracellular biotinamide J Comp Neurol 1995 358 63 78 10.1002/cne.903580104 7560277

[B50] Luo P Zhang J Yang R Pendlebury W Neuronal circuitry and synaptic organization of trigeminal proprioceptive afferents mediating tongue movement and jaw-tongue coordination via hypoglossal premotor neurons Eur J Neurosci 2006 23 3269 3283 10.1111/j.1460-9568.2006.04858.x 16820017

[B51] Wang N May J Peripheral muscle targets and central projections of the mesencephalic trigeminal nucleus in macaque monkeys The Anat Rec 2008 291 974 987 10.1002/ar.20712 PMC2859174 18461596

[B52] Somana R Kotchabhakdi N Walberg F Cerebellar afferents from trigeminal sensory nuclei in the cat Exp Brain Res 1980 38 57 64 10.1007/BF00237931 7351228

[B53] Billig I Yatim N Compoint C Buisseret-Delmas C Buisseret P Cerebellar afferences from the mesencephalic trigeminal nucleus in the rat Neuroreport (Somatosensory System and Pain) 1995 6 2293 2296 10.1097/00001756-199511270-00006 8747139

[B54] Szentàgothai J Anatomical considerations of monosynaptic reflex arcs J Neurophysiol 1948 11 445 453 18883882 10.1152/jn.1948.11.5.445

[B55] Mizuno N Sauerland EK Trigeminal proprioceptive projections to the hypoglossal nucleus and the cervical ventral gray column J Comp Neurol 1970 139 215 226 10.1002/cne.901390205 5422532

[B56] Zhang J Luo P Pendlebury WW Light and electron microscopic observations of a direct projection from mesencephalic trigeminal nucleus neurons to hypoglossal motoneurons in the rat Brain Res 2001 917 67 80 10.1016/S0006-8993(01)02911-0 11602230

[B57] Zhang J Pendlebury WW Luo P Synaptic organization of monosynaptic connections from mesencephalic trigeminal nucleus neurons to hypoglossal motoneurons in the rat Synapse 2003 49 157 169 10.1002/syn.10227 12774300

